# Thermo-Mechanical Recyclability of Additively Manufactured Polypropylene and Polylactic Acid Parts and Polypropylene Support Structures

**DOI:** 10.3390/polym15102291

**Published:** 2023-05-12

**Authors:** Niko Nagengast, Christian Bay, Frank Döpper, Hans-Werner Schmidt, Christian Neuber

**Affiliations:** 1 Chair of Biomechanics, Faculty of Engineering, University of Bayreuth, Universitaetsstrasse 9, 95447 Bayreuth, Germany; 2 Research Center for Additive Innovations, University of Bayreuth, Universitaetsstrasse 30, 95447 Bayreuth, Germany; 3 Chair of Manufacturing and Remanufacturing Technology, Faculty of Engineering, University of Bayreuth, Universitaetsstrasse 9, 95447 Bayreuth, Germany; 4 Chair of Macromolecular Chemistry, Faculty of Natural Science, University of Bayreuth, Universitaetsstrasse 30, 95447 Bayreuth, Germany; 5 Bavarian Polymer Institute, University of Bayreuth, Universitaetsstrasse 30, 95447 Bayreuth, Germany

**Keywords:** thermo-mechanical recycling, additive manufacturing, polymer characterization, Fused Filament Fabrication (FFF), extrusion-based 3D printing

## Abstract

Polymers have a reputation for several advantageous characteristics like chemical resistance, weight reduction, and simple form-giving processes. The rise of additive manufacturing technologies such as Fused Filament Fabrication (FFF) has introduced an even more versatile production process that supported new product design and material concepts. This led to new investigations and innovations driven by the individualization of customized products. The other side of the coin contains an increasing resource and energy consumption satisfying the growing demand for polymer products. This turns into a magnitude of waste accumulation and increased resource consumption. Therefore, appropriate product and material design, taking into account end-of-life scenarios, is essential to limit or even close the loop of economically driven product systems. In this paper, a comparison of virgin and recycled biodegradable (polylactic acid (PLA)) and petroleum-based (polypropylene (PP) & support) filaments for extrusion-based Additive Manufacturing is presented. For the first time, the thermo-mechanical recycling setup contained a service-life simulation, shredding, and extrusion. Specimens and complex geometries with support materials were manufactured with both, virgin and recycled materials. An empirical assessment was executed through mechanical (ISO 527), rheological (ISO 1133), morphological, and dimensional testing. Furthermore, the surface properties of the PLA and PP printed parts were analyzed. In summary, PP parts and parts from its support structure showed, in consideration of all parameters, suitable recyclability with a marginal parameter variance in comparison to the virgin material. The PLA components showed an acceptable decline in the mechanical values but through thermo-mechanical degradation processes, rheological and dimensional properties of the filament dropped decently. This results in significantly identifiable artifacts of the product optics, based on an increase in surface roughness.

## 1. Introduction

Additive Manufacturing (AM) is an advanced manufacturing process characterized by layer-by-layer deposition of a variety of different materials (polymers, ceramics, metals, composites) and shapes of used materials (powder, liquid, sheet, filament). Based on this “bottom-up” approach the AM process is able to realize complexly individualized, technological, functional, and lightweight product design [1,2,3,4]. Fused Filament Fabrication (FFF), one of the most popular techniques of AM, contains the melting, extruding, and positioning of a molten thermoplastic material incrementally layer-by-layer to create a three-dimensional object [5]. Due to the advantages in product and process design, the general AM market is forecast to reach an overall volume of 100 billion US-$ between 2031 and 2044 [6]. Apart from the financial benefits, Gebler et al. predicted a reduction of the global energy demand through AM by 5% [7]. Including improved resource efficiency, extended product life, and reconfigured value chains [8], several studies were investigated to explain the possibilities of AM and sustainability [9,10,11]. Considering the steady rise of consumer and industrial practitioners [12] paired with general challenges [13] of producing the preferred outcome, it is arguable how far the sustainability aspect suits the present AM-process cycle in terms of raw material consumption and waste generation. 

Taking the estimated 12,000 Mt of overall plastic waste covered in landfills by 2050 [14] and the low global recycling rate (approx. 14%) of polymer End-of-Life (EoL) products [15] into account, it is essential to identify appropriate recycling techniques and methods for any polymer-based product manufacturing process. In general, the recycling options are subdivided into primary, secondary, tertiary, and quaternary recycling [16,17,18,19]. Through economic and technological practicability [20], thermomechanical recycling is considered the most common approach for polymer recycling [21,22]. A systematical execution design is proposed by Hopewell et al. [23]: collection, sorting, size reduction and cleaning, further separation, and reproduction. 

Considering the holistic recycling supply chain, various challenges regarding execution practicability appear: The first bottleneck relates to the logistical difficulties in the collection and sorting process of municipal waste streams. Due to the mixture of multiple varieties and complex sorting processes, the goal of obtaining a single plastic material turns out highly investment- and technology-driven [24,25]. Another aspect is the degradation process of the polymer’s microstructure during its service lifetime influenced by the environmental impact and the thermo-chemical wear of the user. Finally, there is a lack of knowledge on how the recycling process, here thermo-mechanical recycling, influences the polymer microstructure and accordingly the preparation of high-quality multiple-life raw materials [26,27,28,29].

With the rise of FFF, a diversity of thermoplastic filaments was developed and tailored for this AM technique: Acrylonitrile butadiene styrene (ABS), Polylactic Acid (PLA), Poly(Ethylene Glycol) Terephthalate (PETG), Polyurethanes (PURs), Polyamide (PA), Polycarbonate (PC), Polypropylene (PP), etc. [30]. The monomer of PLA is usually produced by fermentation processes of natural resources like starch and corn. The PLA used in AM is polymerized yielding a semi-crystalline polyester with a very low degree of crystallinity. Tailored PLA grades are used in a variety of applications such as the packaging, medical, textile, and automotive industry [31,32,33,34]. Furthermore, non-toxicity, biodegradability, low cost, and easy processing characteristics [35,36] have led to a steadily rising interest in FFF [30]. PP is a semicrystalline, non-polar, and typically petroleum-based polyolefin. It is generated through the polymerization of propene, a side product of ethene production [37]. Due to its variety of beneficial characteristics, it covers a broad field of applications in the electronic, packaging, medical, and lightweight industrial sectors [38,39,40,41]. These advantages make it one of the most demanded polymers globally [42]. However, polypropylene produced into filaments or powder for additive manufacturing is still rare, and so far, only non-ideal PP materials for FFF are known [43,44,45]. 

In addition to material properties, a variety of manufacturing settings are responsible for an appropriate result of a 3D printed part. Kechagias et al. proposed a multitude of parameters [46] regarding surface quality and dimensional accuracy in terms of signal (i.e., part orientation, layer thickness, nozzle temperature), control (i.e., material choice, nozzle diameter) and noise parameters (i.e., environmental conditions, motor system). In another study [47] they investigated infill density, raster deposition angle, nozzle temperature, printing speed, layer thickness, and bed temperature via a Design of Experiment (DOE) on PLA. They identified the raster deposition angle, nozzle temperature, printing speed, and infill density significantly impact the mechanical properties of 3D-printed PLA parts. The effect on interlayer properties caused by the rheology, crystallization, and processing of PLA and PP thermoplastics was investigated by Arit et al. [48].

Several studies [49,50,51,52] were carried out describing extrusion-based recycling in general and also of PLA and PP [53]. Additively manufactured PLA shows a significant decrease in rheological and mechanical parameters with multiple recycling cycles with and without the addition of virgin raw material [54,55,56,57,58]. PP, as a relatively new and rare FFF material, is undertaken a variety of studies for thermo-mechanical recycling in AM context [59,60,61,62,63] and without [64,65,66]. The results demonstrate the feasibility of recycled polypropylene, even after multiple recycling cycles. Also, some studies [67,68] implemented waste material as an input option for AM, which complements the recycling possibilities of industrial unmixed materials on a broader scale.

In terms of usability in the product manufacturing process via AM, the filament, processing properties, and end product regarding a wide range of performance parameters should be analyzed and evaluated. Badia and Amparo [69] proposed a magnitude of characterization methods: Reprocessing simulation, service life simulation, structural assessment, morphological characterization, rheological, thermal, and mechanical properties, monitoring, molecular weight, and application-driven characterization. Additional studies [70,71] have been conducted to define test protocols and quality improvements for material recycling.

**Figure 1:**
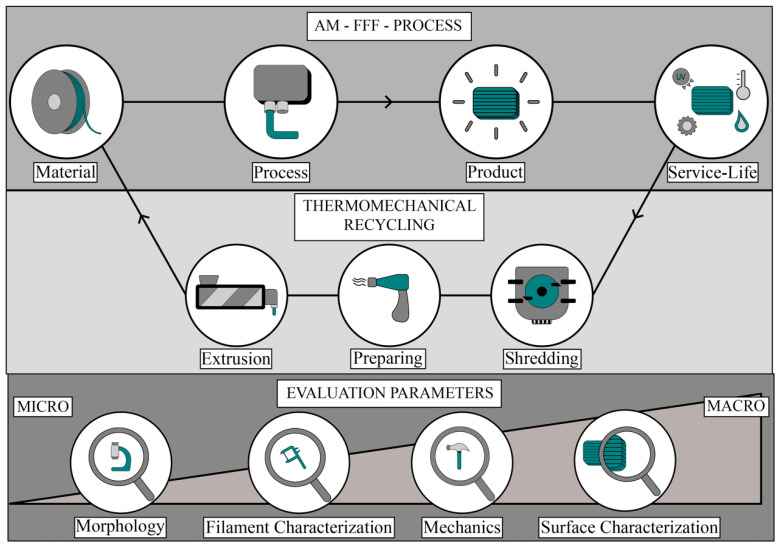
Simplified process strategy of recycling purposes in extrusion-based Additive Manufacturing (AM).

However, most of the published work has focused particularly on the potential change in material properties regarding recycling and is concerned with the direct recycling of just before extruded single filament material. However, this work focuses on the material recycling of FFF printed parts made of different materials and evaluates for the first time the change in material properties after a service life simulation based on selected parameters (Figure 1). 

## 2. Materials and Methods

The process starts with the AM process to produce 3D printed test specimens called products, and these parts are aged through a simulated use phase called service-life (see Figure 1). After that, the aged parts were thermos-mechanically recycled and filaments were prepared for the AM process of 3D-printed parts using recycled materials. The evaluation comprised the characterization of morphological, filament-based, mechanical, and product aesthetical parameters. 

All virgin filaments featured a diameter of 1.75 mm. The red-colored Premium PLA Raise3D (Costa Mesa, CA, USA) was utilized for the AM of all PLA objects. Additionally, a PVA Natural support filament, produced by FormFutura (Nijmegen, The Netherlands) was used for the complex geometry of an impeller blade. The PVA support material is developed to be dissolved in a water bath and the obtained liquid polymer solution is not applicable to the thermomechanical recycling process. The PP and PP support material was provided by PPprint GmbH (Bayreuth, Germany). The used filaments consisted of the P-filament 721 natural and P-support 279 natural according to the object and support geometry.

**Figure 2:**
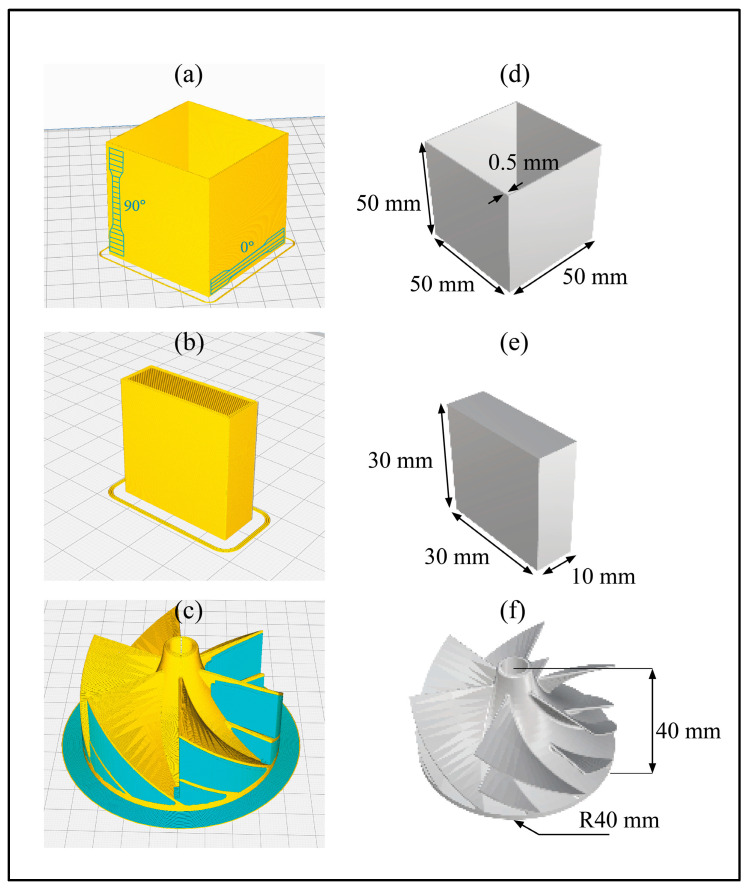
Sliced geometry and CAD drawing with geometric dimensions: (**a**) Squared tube for tensile test specimen fabrication by punching out dog-bones of selected orientations (sliced), (**b**) simple cuboid coin geometry (sliced), (**c**) complex impeller blade geometry with a support structure (CAD), (**d**) squared tube for tensile test specimen fabrication (CAD), (**e**) simple cuboid coin geometry (CAD), (**f**) complex impeller blade geometry (CAD).

An extrusion-based AM machine *Raise3D E2* was used for the printing process utilizing two filament extruders. The build volume is for the dual printing (295 × 240 × 240) mm^3^. Due to improving adhesion reasons, the build surface P-surface 141 (PPprint GmbH) was attached to the top of the printing bed. Both extruders used a nozzle diameter of 0.4 mm. After preliminary printing tests to optimize the printing conditions, the general printing parameters were equally applied for all PP and PLA parts. Taking the complex structure into account, the temperature of PVA was set to 190 °C and the horizontal offset of the supporting structure was increased because of weak bonding properties between PLA and used PVA. The printing parameters for the different printed parts (see Figure 2) are shown in Table 1. All three geometries were printed with virgin as well as recycled materials, except for the PVA filament. Additionally, only the PLA and PVA filaments were dried for 24 h under 60 °C in a Thermo Scientific VT 6060–P drying oven from “Thermo Electron LED GmbH” (Langenselbold, Germany) under vacuum. The vacuum was generated by a VacuUUbrand RD vacuum pump from “Rudolf Brand GmbH+Co. KG” (Wertheim, Germany). The punched-out dogbone specimens for the tensile tests followed the geometry conditions of DIN ISO 527 S3A (Figure 2, top).

**Service-life simulation.** The service life of the additively manufactured cuboids was simulated by accelerated aging according to sterile barrier systems for medical polymer devices. The process was adopted from ASTM F1980-16 [72]. The simulation process was realized for two weeks at 60 °C and 7.8% air humidity using a Carbolite Convection Drying Cabinet PF 120/2416 *CG* from “Rettberg Gebr. GmbH” (Göttingen, Germany). The accelerated aging process approximately corresponds to about half a year of use at room temperature aging. The service life of half a year was chosen because additively manufactured parts are often prototypes or short-lived parts. 

**Recycling process.** The cuboids made from PLA, PP, and PP support filament were transferred into the recycling process after the accelerated aging. An SHR3D IT shredding machine, with a three-stage crushing appliance from “3devo B.V.” (Utrecht, The Netherlands) was used to shred the single-variety material into flakes. The implementation of a metal sieve at the end controlled the maximal diameter size of 4 mm. Before extrusion, the PLA flakes were processed through the same drying process mentioned earlier for FFF printing. An Extruder Next 1.0–advanced from “3devo B.V.” was used to produce the recycled filaments of the investigated polymers. The process was carried out identically for the PLA, PP, and PP support filaments. The rotational speed of the single screw was set to 3 rpm. The temperature of the four heat zones was controlled at 140 °C for the first one (filler tube) and 180 °C for the second to fourth one (extruder nozzle). The cooling of the extruded filament was set to 25% of the maximal fan operation. In order to the avoidance of too high filament diameters, the filament diameter variable was set to 1.73 mm. 

**Morphology.** For the characterization of the crystalline behavior of the polymeric materials, the crossed polarized light microscope (PLM) “Inverse Nikon Diaphot” (Minato, Japan) with a resolution of 1:50 was used. All the virgin and recycled filaments (PLA, PP, PP Support) were cut into about 10 µm thin slices by a Microtome (Leica, Wetzlar, Germany) cutting tool. To reduce stress-related birefringence the thin slices were annealed at 70 °C for 30 min. The pictures were recorded at 0° orientation to the cutting direction.

**Filament and material characterization.** Two characteristic parameters of the extruded filaments were investigated: (a) geometric aspects (diameter, ovality) and (b) melt flow. 

The geometric parameters were measured by a laser measurement unit from “Zumbach” (Orpund, Switzerland). For every investigated filament at least 50 m were measured and the average values were reported. Aside from the mean and standard deviation, especially the obtained local Minima and Maxima of the diameter influence the quality of a print in terms of sufficient material deposition and the risk of nozzle clogging. The melt flow of all polymers was investigated via the DIN EN ISO 1133 of the Melt Flow Rate (MFR) with the assistance of a Rheo Meltflixer from “SWO Polymertechnik” (Krefeld, Germany). A sample size of four runs per polymer filament was executed. Once more, the PLA filament was dried utilizing the above-described parameters. One run contained seven grams of pelletized polymer filament and allowed five measurements of MFR. The used weight load was 2.16 kg and a temperature of 230 °C was applied. The MFR measurement is an indicator for the averaged molecular weight, chain branching, and branching characteristics–like the degree of distribution of branching [72].

**Mechanical testing.** The tensile specimens were punched out of the 0.8 mm thick side walls of the corresponding square tubes (Figure 2) utilizing a hydraulic sample punch from “Coesfeld” (Dortmund, Germany). 10 specimens in 0°, as well as 90° alignment to the manufacturing direction of every investigated polymer material, were tested. According to post-crystallization processes, the PP and PP support samples rested for 48 h. The PLA specimens were packed into airtight packaging for the same time period. 

Implementing the guidelines of DIN EN ISO 527, the tensile test was carried out with an Instron 5565 from “Instron” (Norwood, MA, USA), using a load cell of 1 kN and a non-contacting video extensometer for the tensile strain measurement. The test parameters were set to 0.1 mm/min and 5 mm/min regarding the elastic and viscoelastic/plastic intervals. The tensile testing was performed at 25 °C and an air humidity level of 50%. 

**Figure 3:**
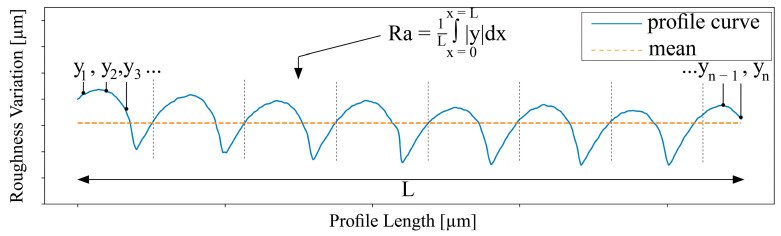
Exemplary profile curve of PP measured by Dektak 150 Surface Profiler from Bruker.

**Surface characterization.** The surface texture of an AM-produced part indicates the processability of the material and the fulfillment of application-specific requirements. Therefore, the surface properties (Figure 3) of the virgin and recycled PLA- and PP-printed cuboids were investigated by a stylus profilometer Dektak 150 Surface Profiler from “Bruker” (Billerica, MA, USA). The averaged roughness Ra results from the integration of the measured points y_n_ (‘height value’) in consideration of the total length L of the measured profile. Each measured y-value corresponds to an x-value depending on its position along the profile length. 

Finally, the integration result is averaged through the profile length L. 

The length of 1500 µm in the x and y direction of the y-z plane (30 mm × 30 mm plane of the cuboid; Figure 2) was scanned by 30 profiles. The direction of the measurement followed orthogonal to the printing direction. One profile measurement took 30 s. The resolution was set to 0.167 µm/sample, whereas the Y resolution was set to 50 µm/profile. The needle was pressed onto the surface with a force of 3 mg. The measurement range covered 524 µm. 

## 3. Results

This section may be divided into subheadings. It should provide a concise and precise description of the experimental results, their interpretation, as well as the experimental conclusions that can be drawn.

### 3.1. Fabrication and Recycling Process

**Figure 4:**
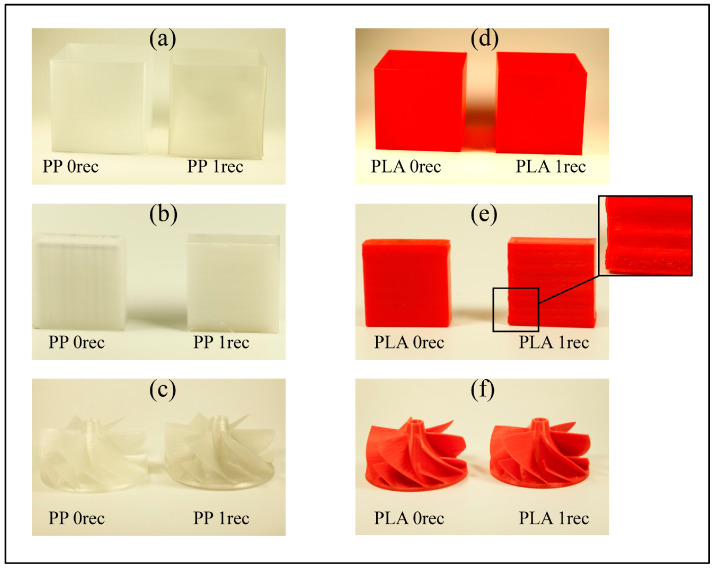
Investigated products: (**a**) Squared tube PP and PP recycled, (**b**) cuboids PP and PP recycled, (**c**) complex impeller blade geometry PP and PP recycled, (**d**) squared tube PLA and PLA recycled, (**e**) cuboids PLA and PLA recycled, (**f**) complex impeller blade geometry PLA and PLA recycled.

The material recycling process was successfully executed by the production of applicable corresponding filaments which were used for the extrusion-based AM of various geometries (square tube, cuboid, impeller). For the characterization, virgin (called 0rec) and recycled (1rec) materials were compared to each other (see Figure 4). Considering the optical appearance, a marginal difference in the natural color of the part printed from PP is observable. A light gray coloration of the recycled PP filament was observed after the shredding of the cuboids. According to its low glass transition temperature (T_g_), the PP cuboids became soft during the shredding and smeared, resulting in the shredding process taking longer. Due to the higher T_g_ of the PP support material, this effect did not occur with the support material.

During the filament extrusion, the material feed of the PP and PP support flakes varied because the shredded and cross-grained flakes did not exhibit sufficient pourability. The resulting discontinuous feeding resulted in an irregular diameter of the obtained recycled polyolefin filaments and particularly in an increased ovality of the recycled PP filament (see Table 3 and Table 4). Therefore, these patchy filaments were pelletized and re-extruded.

Comparing the PLA prints, the development of ripples at the recycled PLA geometries can be observed. This can be explained by the decreasing viscosity described later in Section 3.3 filament and material characterization. All filaments used for the support structures (PVA, virgin, and recycled PP support) fulfilled the purpose to obtain a complex geometry (impeller) and could be removed (PVA: dissolution in water; PP support: break-away at elevated temperature) without complications. Due to a weak bonding of the PLA-PVA-PLA layers between the bottom of the impeller and the blades, the horizontal offset had to be increased so that the PVA support structure was linked to the PVA raft of the impeller. The PLA impeller was conditioned for 24 h and 60 °C in a vacuum oven setup (see also Section 2 Materials and Methods) after the PVA dissolution and thus removal in the water bath. 

### 3.2. Morphology

**Figure 5:**
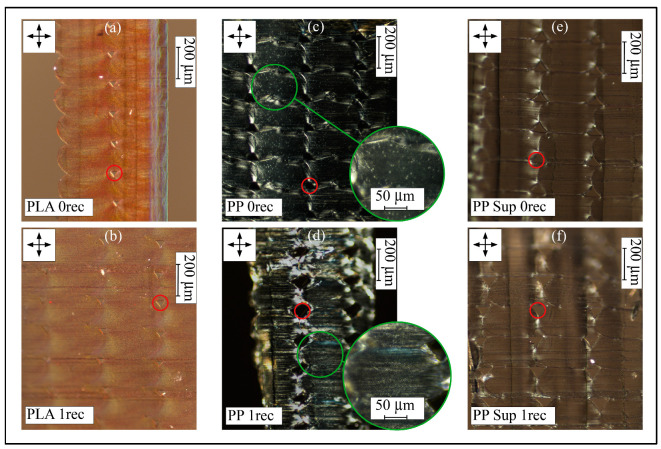
Cross-polarized light microscopic images of thin slices of all investigated polymers: (**a**) PLA, (**b**) PLA recycled, (**c**) PP, (**d**) PP recycled, (**e**) PP support, (**f**) PP support recycled.

The investigation of the crystalline behavior was realized by the observation of thin polymer slices (PLA, PP, PP support) in their virgin and recycled states using a cross-polarized light microscope. Figure 5 shows cross-polarized light microscopic images of corresponding thin slices of the polymer strands, where the typical oval shape of the printed filament is visible. The small gaps (red circles) between the strands are mainly influenced by filling parameters such as applied nozzle and filament diameter and extrusion volume. The shown strands were performed layer-by-layer from bottom to top. The general brightness difference describes crystalline (bright) and amorphous (dark) regions in the polymer. By cutting those thin slices, stress birefringence can occur in the form of very bright areas, even if it has been reduced by annealing. 

Referencing the geometrical shape of the staged single additively manufactured strands, there is no clear obvious difference between the virgin and the recycled prints in all three cases. Considering multiple strands and layers, the gap size (red circles in Figure 5) slightly increased towards the recycled prints. Due to the lower degree of crystallinity in the PLA- and the PP support materials (see Table 2), less or no significant bright spots, as a feature of crystalline behavior, appear. Again, no important differences in these materials can be observed after recycling.

Only the first heating curves were used for the calculation of the degree of crystallinity according to Equation (1):

(1) $$X_{C}=\frac{\Delta H_{m}}{\Delta H_{m}^{100}}\times 100\%$$

where $\Delta H_{m}$ is the measured melting enthalpy (minus eventually measured enthalpy of cold crystallization) and $\Delta H_{m}^{100}$ is the melting enthalpy of the corresponding 100% crystalline polymer material as known from the literature [73,74].

The additively manufactured strands of the virgin PP materials are patterned by multiple bright spots. This morphological characteristic appears when the polymer solidifies after melting due to the formation of crystals. The strands manufactured with the virgin PP filaments show larger and brighter spots while the strands printed with the recycled PP filament show more uniformly sized bright spots (green circles). In this context, isothermal crystallization measurements were realized by DSC measurements showing a faster nucleation formation step for the recycled PP material (see Supplementary Materials, Figure S1). This behavior may originate from contamination during the PP shredding (light gray coloration), while the degree of crystallization is approximately the same for virgin and recycled PP materials.

### 3.3. Filament and Material Characterization

Table 3 shows the values of melt flow rate (MFR) measurements of the virgin and recycled polymers (PLA, PP, PP Support), regarding ISO 1133. The MFR of the PLA filament significantly increased with the recycling process. The relative $\Delta {MFR}_{PLA}{=MFR}_{i+1}{/MFR}_{i}$ resulted in a rise of 83.3%. Contrary to the PLA filament, the MFR of the PP and PP Support filament did not change evidently after the remanufacturing process, with $\Delta {MFR}_{PP}=11.25\%$ and $\Delta {MFR}_{PP\;Sup}=0$ and their corresponding deviations of ±0.7–1.6 g/10 min for the PP and ±0.1–0.2 g/10 min for the PP Support filament. 

The thermal and mechanical energy exposure during the service-life simulation, the shredding, and extrusion can lead to a degradation of the polymer chains, additional branching, and crosslinking [75]. This affects the melt flow properties and therefore the part quality [76]. Thus, the high MFR increase for the recycled PLA filament can be explained through various changes in their polymer chain length and be pointed out at the product scale through the ripples visible in Figure 4 for PLA 1rec. Considering the negligible MFR changes in both recycled polyolefin filaments, the thermal-mechanical treatment, due to the recycling process, did not show any significant impact on the melt flow properties of these polymers.

**Table 3:** Measured MFR values of investigated polymers.

	MFR Values [g/10 min; 2.16 kg; 230 °C]					
	PLA 0rec	PLA 1rec	PP 0rec	PP 1rec	PP Sup 0rec	PP Sup 1rec
MAX	37	56	14	9	11	11
MIN	23	54	8	7	10	10
MEAN	30	55	9	8	10	10
STDV	3.6	1.0	1.6	0.7	0.2	0.1

A homogeneous filament diameter allows a constant material feed through the nozzle to extrude a uniformly printed object. Industrial standards demand a maximum tolerance of 1.75 mm ± 0.05 mm, whereas other sources expand the criteria up to an interval of 1.70 mm–1.825 mm [77], or even 1.75 ± 0.1 mm [78]. As mentioned before, especially the minimum and maximum values can result in less material extrusion and thus poor surface properties, or in the worst case, a clogging of the nozzle.

Regarding the industrial standard, only the virgin PLA, PP, and PP Support filaments fulfill the tolerances with 1.76 ± 0.02 mm, 1.72 ± 0.04 mm, and 1.73 ± 0.02 mm. Expanding the tolerance, the one-time extruded PLA and both double-extruded PP (PP 1rec_2extr) and PP Support filament (PP Sup 1rec_2extr) satisfy processability parameters (Table 4). The double extrusion of the polyolefin polymers was necessary, as the irregular feeding of the obtained flakes from the shredding resulted in non-uniform filament diameters for the first extrusion. Here, pelletizing and re-extrusion allowed regular feeding. In this context, a decrease in the desired tolerance of the PLA filament diameter after a second re-extrusion was observed. In contrast, the second extrusion of the polyolefin filaments decreased the diameter variation and allowed the production of filaments that meet industry standards. This manifests the hypothesis of the degradation of the PLA due to multiple thermo-mechanical treatments. 

**Table 4:** Measured filament diameter values of investigated polymers.

	Filament Diameter Values [mm]								
	PLA 0rec	PLA 1rec_1extr	PLA 1rec_2extr	PP 0rec	PP 1rec_1extr	PP 1rec_2extr	PP Sup 0rec	PP Sup 1rec_1extr	PP Sup 1rec_2extr
MAX	1.78	1.94	2.11	1.76	2.04	1.81	1.75	2.50	1.79
MIN	1.75	1.54	1.39	1.68	0.24	1.63	1.72	1.30	1.60
MEAN	1.76	1.72	1.71	1.72	1.65	1.69	1.73	1.69	1.67
STDV	0.01	0.06	0.12	0.01	0.22	0.03	0.01	0.13	0.03

In addition to the diameter, the ovality values of the filaments influence the quality of the printing process. Spoerk et al. [79] recommended an acceptable tolerance of up to 0.05. All virgin and recycled filaments fulfilled this mark, except the PP filament after the first extrusion (Table 5). 

### 3.4. Mechanical Testing

Specimens for both, virgin and recycled filaments, were tested in 0° direction and 90° direction in x-y direction of movement of the 3D printer extruder regarding ISO 527. The 0° direction specimens provide values that almost correspond to those of injection-molded specimens and should serve only as a reference (see Supplementary Materials, Tables S1–S3). Therefore, characterizing the mechanical performance of the parts regarding the layer bonding (strength in orthogonal direction) of the single strains is of importance and realized here by 90° direction measurements (for more details see Supplementary Materials, Figure S2). 

**Figure 6:**
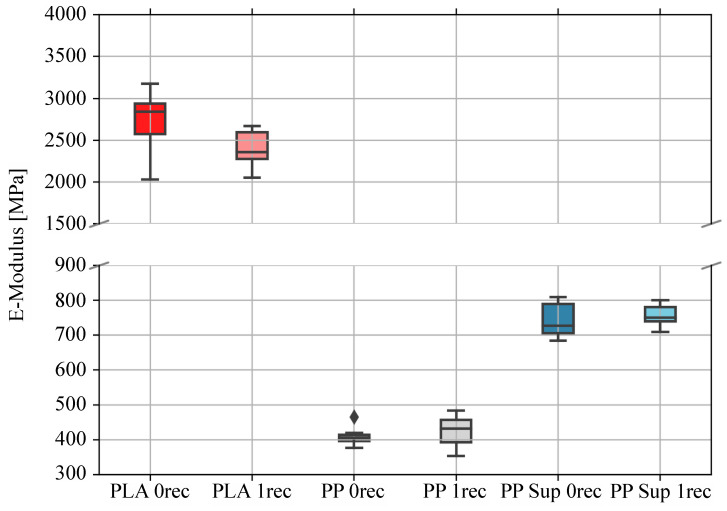
Graph of E-Moduli of 3D printed specimens in 90° direction to the x-y direction of movement of the 3D printer extruder. (Box-Whisker Plot: median = horizontal line, box (interquartile range (IQR)) = upper border (75th percentile, Q3), lower border (25th percentile, Q1), Max = upper T-beam (Q3 + 1.5 × IQR), Min = lower T-beam (Q1 − 1.5 × IQR), diamond = outliers.).

The stress-strain correlation in the elastic region is described by the E-modulus and signifies the stiffness of a material. As shown in Figure 6 and Table 6 PLA is located in a far stiffer property region than PP and PP support. Consequently, there will be only a comparison of the virgin and recycled materials of the individual polymer types.

Considering Table 6, the E-modulus of the virgin PLA amounts to 2724 ± 391 MPa and the recycled material to 2404 ± 214 MPa. This represents a decrease from the mean of 11.75%. The values of PP are 410 ± 27 MPa for the virgin and 424 ± 66 MPa for the recycled material. A similar tendency is visible for the E-modulus of the PP support material, representing 743 ± 54 MPa for the virgin and 756 ± 34 MPa for the recycled material. The slight increase of the recycled PP material accounts for 3.4%, whereas the values of the recycled support material enhance to 1.7%.

**Figure 7:**
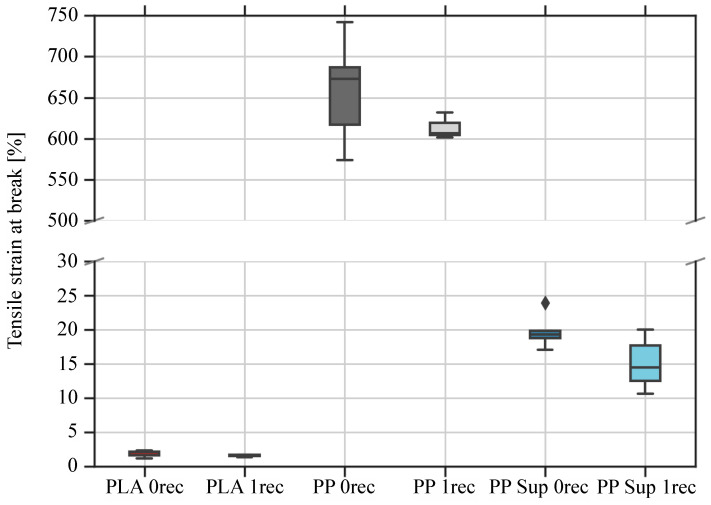
Graph of tensile strain values of 3D printed specimens in 90° direction to the x-y direction of movement of the 3D printer extruder.

PLA is a stiff but brittle material, whereas PP is considered a more ductile thermoplastic material (Figure 7 and Table 7). The PP support material can be described as in-between, fulfilling enough stiffness and deflection properties to maintain adequate structural support properties for the print (see Figure 6 and Figure 7). For all three polymers, a decline in the tensile strain property is indicated because of the recycling process. 

The PLA tensile strain values were reduced from 1.9 ± 0.4% to 1.6 ± 0.1% resulting in a decline of 15.8%. Furthermore, the tensile strain of the PP material dropped from 659 ± 55% to 614 ± 16% by a percentage of 6.8%. For the support material, the values decreased from 20 ± 2.6% for the virgin polymer to 15 ± 3.7% for the recycled polymer. This leads to a regression of 25%.

**Figure 8:**
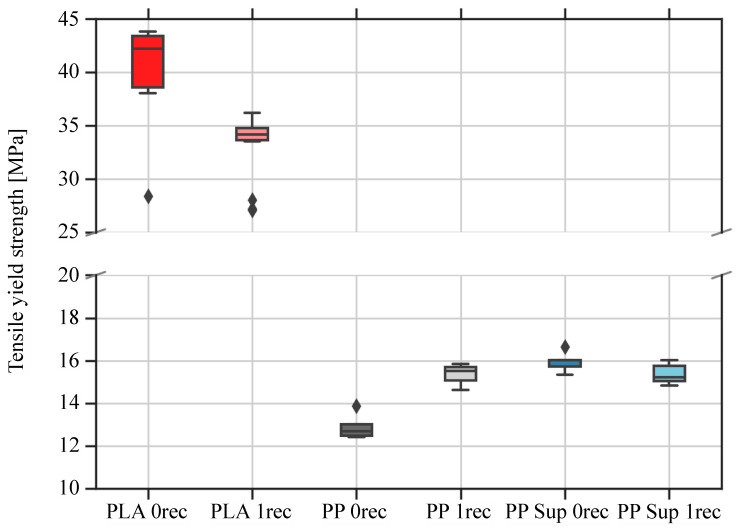
Graph of tensile stress values of 3D printed specimens in 90° direction to the x-y direction of movement of the 3D printer extruder.

Figure 8 shows the graph of tensile stress values of 3D printed specimens in 90° direction to the x-y direction of movement of the 3D printer extruder, where a decline of the PLA and PP support material and an increase of the PP material is observable (for more details see also Table 8).

Summarizing Figure 8 and Table 8, the tensile stress reduction of the recycled PLA filament compared to virgin material was 17.5% on average. The tensile stress of the PP filament increased from 13 ± 0.5 MPa to 15 ± 0.6 MPa by 15.4%, whereas the support material marginally declined the values from 16 ± 0.5 MPa to 15 ± 0.5 MPa by 6.25%.

The mechanical properties of materials depend on various factors at the molecular level. In the case of polymeric materials, the macromolecular level is an additional important factor. All three investigated mechanical parameters are closely related to changes in inter- and intramolecular interactions influenced by the length of polymer chains and their architectures, glass transition temperature (chain segment stiffness), and crystallinity (degree, type, distribution, size). Additionally, chain entanglement plays an important role in the tensile strain and stress attributes. This correlates to the molecular weight and is, therefore, an indicator for manifold degradation and crosslinking processes during thermo-mechanical recycling [80,81,82].

As discussed before, all three investigated mechanical test values of the PLA material clearly decrease from virgin to recycled material. This result is consistent with various other studies in the field [52,53,55,56]. The degradation processes can be explained through the hydrophilic character and the thermal hydrolysis of PLA [83]. The decrease is therefore due to the recycling process, during the service-life simulation, thermal conditioning via the vacuum oven before each extrusion, the filament extrusion itself, and the AM process. The finding is supported by Yu et al. [84], who pointed out that temperatures around 180–220 °C typically lead to degradation processes in the polymer chain. 

On the other hand, none of the polyolefins showed significant deterioration in mechanical properties. These findings are consistent with the studies of Spoerk et al. [58] and Vidakis et al. [60]. Through its hydrophobia, the missing of a functional group, and the presence of various additives (i.e., heat stabilizer and UV stabilizer), degradation processes are marginal and recycling processes up to 14 times [59] without major modifications may be possible.

The maximum tensile stress and Young’s modulus of printed parts can be increased by optimized post-annealing processes, indicating an increased interlayer bonding and a reduction of residual stresses in the annealed 3D printed parts. However, the typically observed reduction in toughness shown by a decreased elongation at break and the extra post-treatment process requires a case-by-case assessment [85].

### 3.5. Surface Characterization

**Figure 9:**
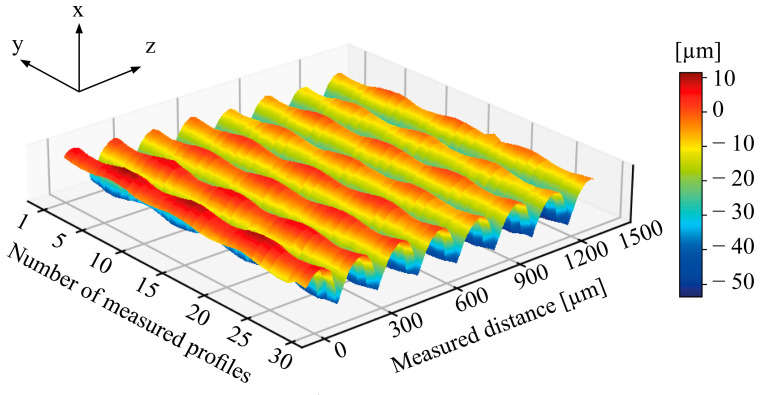
Exemplary surface profile of a virgin PLA cuboid. 30 profiles lined up side by side visually as a 3D-plotted surface profile.

A solid 3-dimensional surface can be described through its roughness, waviness, lay, and flaws [86], whereas micro-roughness seemed to be the appropriate evaluation of 3D printed cuboids. Figure 9 shows 30 profiles lined up side by side visually as a 3D-plotted surface profile of one of the investigated cuboids, with the peaks and valleys along the *z*-axis, the single profiles along the *y*-axis, and the measured roughness along the *x*-axis.

The here shown surface roughness results are the averaged sum of measured profiles, according to the equation:

(2) $$Ra=\frac{1}{L}\;\overset{x=L}{\underset{x=0}{\int }}\left|y\right|dx$$

As shown in Table 9, the average surface roughness of the virgin towards the recycled PLA part increased from 13.1 μm to 17.7 μm. That represents an increase of 35.1%. The difference between the PP parts is less than 1%.

Having measured the surface roughness of printed parts orthogonally with a layer height of 0.2 mm and a nozzle diameter of 0.4 mm, the values correspond to the value dimensions (9.1–32.16 μm with a 0.2–0.5 mm nozzle diameter in 90° measurement direction) of Alsoufi & Elsayed [87] that have tested 0°/45°/90° with various layer heights and nozzle diameters. Similarly, Chaidas et al. reported a surface roughness for parts printed from PLA between 13 and 22 µm and reported that surface roughness decreased with increasing nozzle temperature [88]. The significant increase in the PLA values for the recycled material can be explained by the increased MFR-values of PLA 1rec in Table 3 and the output optics of Figure 4. The lower viscosity of the recycled material seems to result in a more irregular flow of material during the layer deposition, creating increased “ripples”.

**Figure 10:**
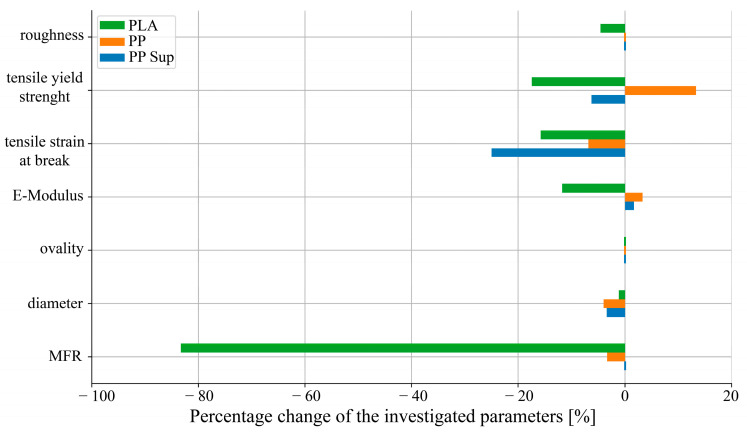
Overall comparison of the virgin and recycled polymers by the percentage change in the parameters investigated.

**Summary.** All the polymers studied achieved some degree of recyclability, although the product designer has to clarify whether certain material properties can meet critical requirements. Figure 10 shows the percentage change in investigated parameters of the virgin (0rec) compared to the recycled (1rec) polymer materials. Tensile yield stress, tensile strain at break, and E-modulus were normalized against the highest value, whereas the roughness and the ovality values were set against the ideal (“zero”) and their difference was normalized against each other. The MFR value of each virgin polymer material was always considered an optimal reference and normalized against the MFR value of the recycled polymer. The absolute difference of the filament diameter to the ideal 1.75 mm was normalized and compared against each other.

Figure 10 points out that the properties of the PLA polymer (green) decreased during the recycling process. The significant change of the MFR value to the negative and thus the deterioration of the surface has a detrimental effect on the geometric design of PLA recycled parts. In addition, the decrease (12.5–17.5%) in mechanical properties is a point to consider for a functional part design. The filament characterization, realized by ovality and filament diameter measurements, shows a marginal effect due to the recycling process. The measured values of the PP filament (orange) remain in an acceptable range after thermomechanical recycling. Although a decline of the tensile strain value over 5% is observed, all other properties show deniable or even improved results for PP. These are indicators of the recycling potential of additively manufactured PP parts. 

Similar to PP, the tensile strain of the PP support filament (blue) decreased due to recycling. Nevertheless, the other properties are located in a reasonable range. Besides the property values, the functionality aspect in terms of the supporting, bonding, and removal characteristics was successfully observed for the AM using an impeller geometry. 

## 4. Conclusions

The investigated work implemented for the first time a full thermo-mechanical recycling circle using 3D printed parts with a service-life simulation and re-manufacturing of a bio-based (PLA) and two petroleum-based (PP & PP support) polymers for additive manufacturing. A general approach of property assessment for the comparison of the virgin and the recycling material ranging from micro- to macro-scale was executed. The main results of the study contain the PLA filament showed a decrease in all properties after the recycling cycle. Especially, the MFR value increased significantly. While the PP filament and PP support filament showed an acceptable performance in all categories after the recycling cycle and thus offer the possibility of direct product recycling.

Next to other investigations in the field [59,60], the results demonstrate the qualification of polypropylene and for the first time its polyolefin support material for recycling purposes in AM. In particular, the break-away PP support structure, which can be recycled as a single-grade material after the printing process, can achieve a high recycling potential through suitable operational and logistical measures. On the other hand, PLA faces great challenges for AM to exhibit suitable properties after recycling. However, for a better understanding and qualification further detailed investigation on the degradation behavior of PLA and identification of additives for the stabilization of PLA during recycling are needed.

In terms of PP recycling, the material recycling in AM has to be supported by single-grade material collection and municipal waste management. In this context, the integration of labeling systems for AM materials to characterize recycling content and behavior would be desirable.

The here presented performance characterization of the materials regarding recycling is a mandatory and first step in proving the quality aspects of circular approaches. Nevertheless, a pure performance qualification through recycling does not qualify a material to be sustainable or not. The study proved the feasibility of the investigated thermoplastics for FFF after polymer aging and recycling. All the recycling processes were carried out at a lab scale and in-house with unmodified materials. In terms of industrial scalability bottlenecks like material return logistics, sorting, and cleaning are essential steps to consider. It is also advisable to combine a performance characterization with an environmental and economic assessment to describe the full meaningfulness of recycling.

## Figures and Tables

**Table 1:** Printing parameters of the cuboids, impellers, and square tubes.

Parameters	Cuboid	Impeller	Square Tube
Nozzle temperature [°C]	210	210	210
Bed temperature [°C]	20 (first layer 70)	20 (first layer 70)	20 (first layer 70)
Layer height [mm]	0.2	0.2	0.2
Layer width [mm]	0.4	0.4	0.4
Top solid layers	3	5	1
Bottom solid layers	3	5	1
Infill density [%]	50	25	50
Support infill density [%]		25	
Material flow [%]	100	100	100
Travel speed [mm/s]	20	20	20
Adhesion structure	Skirt	Raft	Skirt

**Table 2:** Degree of crystallinity of investigated materials was determined using DSC values.

Material	ΔH_m_ [J/g]	$\Delta H_{m}^{100}\;[J/g]$	X_C_ [%]
PLA 0rec	2	93.7 [73]	2.1
PLA 1rec	4		4.3
PP 0rec	59	171 [74]	34.5
PP 1rec	57		33.3
PP Sup 0rec	11	-	-
PP Sup 1rec	12		-

**Table 5:** Measured filament ovality values of investigated polymers.

	Filament Ovality Values								
	PLA 0rec	PLA 1rec_1extr	PLA 1rec_2extr	PP 0rec	PP 1rec_1extr	PP 1rec_2extr	PP Sup 0rec	PP Sup 1rec_1extr	PP Sup 1rec_2extr
MAX	0.019	0.024	0.031	0.055	0.562	0.044	0.02	0.035	0.033
MIN	0.002	0.001	0.000	0.015	0.011	0.007	0.003	0.003	0.004
MEAN	0.009	0.008	0.007	0.033	0.097	0.015	0.011	0.014	0.017
STDV	0.003	0.004	0.005	0.005	0.129	0.004	0.003	0.004	0.004

**Table 6:** E-modulus values of 3D printed samples in 90° direction to the x-y direction of movement of the 3D printer extruder (10 specimens per sample).

	90° E-Modulus Values [MPa]					
	PLA 0rec	PLA 1rec	PP 0rec	PP 1rec	PP Sup 0rec	PP Sup 1rec
MAX	3177	2673	467	485	809	800
MIN	2029	2048	377	354	684	709
MEAN	2724	2404	410	424	743	756
STDV	391	214	27	66	54	34

**Table 7:** Tensile strain values of 3D printed samples in 90° direction to the x-y direction of movement of the 3D printer extruder (10 specimens per sample).

	90° Tensile Strain at Break Values [%]					
	PLA 0rec	PLA 1rec	PP 0rec	PP 1rec	PP Sup 0rec	PP Sup 1rec
MAX	2.3	1.8	742	632	24	20
MIN	1.2	1.3	574	602	17	11
MEAN	1.9	1.6	659	614	20	15
STDV	0.4	0.1	55	16	2.6	3.7

**Table 8:** Tensile stress values of 3D printed samples in 90° direction to the x-y direction of movement of the 3D printer extruder (10 specimens per sample).

	90° Tensile Yield Strength Values [MPa]					
	PLA 0rec	PLA 1rec	PP 0rec	PP 1rec	PP Sup 0rec	PP Sup 1rec
MAX	44	36	14	16	17	16
MIN	28	27	12	15	15	15
MEAN	40	33	13	15	16	15
STDV	5.5	3.1	0.5	0.6	0.5	0.5

**Table 9:** Averaged surface roughness values of additively manufactured PLA and PP cuboids.

	Roughness [μm]			
	PLA 0rec	PLA 1rec	PP 0rec	PP 1rec
**R_a_**	13.1	17.7	11.6	11.7

## Data Availability

The data presented in this study are available on request from the corresponding author.

## Supplementary Materials

The following supporting information can be downloaded at: https://www.mdpi.com/article/10.3390/polym15102291/s1, Figure S1: DSC curves of isothermal crystallization measurements of the virgin (PP 0rec) and recycled (PP 1rec) Polypropylene at T = 110°C; Figure S2: Tensile stress-strain curves of investigated polymers in 90°- direction; Table S1: E-Modulus of the investigated polymers in 0°- direction; Table S2: Tensile strain values of investigated polymers in 0°- direction; Table S3: Tensile yield strength values of investigated polymers in 0°- direction.

## References

[1] Schmitt P., Zorn S., Gericke K. Additive Manufacturing research landscape. A literature review. Proceedings of the International Conference on Engineering Design (ICED21).

[2] Ngo T., Kashani A., Imbalzano G., Nguyen K., Hui D. (2018). Additive Manufacturing (3D printing): A review of materials, methods, applications and challenges. Compos. Part B.

[3] Maguire A., Pottackal N., Saadi M., Rahman M., Ajayan P. (2021). Additive Manufacturing of polymer-based structures by extrusion technologies. Oxf. Open Mater. Sci..

[4] Bourell D., Kruth J., Leu M., Levy G., Rosen D., Beese A., Clare A. (2017). Materials for Additive Manufacturing. CIRP Ann..

[5] Rashid A., Koç M. (2021). Fused Filament Fabrication Process: A review of numerical simulation techniques. Polymers.

[6] Tofail S., Koumoulos E., Badyopadhyay A., Bose S., O’Donoghue L., Charitidis C. (2018). Additiv Manufacturing: Scientific and technological challenges, market uptake and opportunities. Mater. Today.

[7] Gebler M., Uiterkamp A.S., Visser C. (2014). A global sustainability perspective on 3D printing technologies. Energy Policy.

[8] Ford S., Despeisse M. (2016). Additive Manufacturing and sustainability: An exploratory of the advantages and challenges. J. Clean. Prod..

[9] Peng T., Kellens K., Tang R., Chen C., Chen G. (2018). Sustainability of Additive Manufacturing: An overview on its energy demand and environmental impact. Addit. Manuf..

[10] Colorado H.A., Velásquez E.I.G., Monteiro S.N. (2020). Sustainability of Additive Manufacturing: The Circular Economy of materials and environmental perspective. J. Mater. Res. Technol..

[11] Kellens K., Baumers M., Gutowski T., Flanagan W., Lifset R., Duflou J. (2017). Environmental dimensions of AM: Mapping application domains and their environmental implications. J. Ind. Ecol..

[12] Streenhuis H.-J., Pretorius L. (2015). Additive Manufacturing or 3D printing and its adoption. IAMOT 2015 Conference Proceedings.

[13] Oropallo W., Piegl L. (2016). Ten challenges in 3D printing. Eng. Comput..

[14] Geyer R., Jambeck J., Law K. (2017). Production, use, and fate of all plastics ever made. Sci. Adv..

[15] Hahladakis J., Iacovidou E. (2018). Closing the loop on plastic packaging materials: What is quality and how does it affect their circularity?. Sci. Total Environ..

[16] Wang M., Liu P., Gu Z., Cheng H., Li X. (2019). A scientometric review of resource recycling industry. Int. J. Environ. Res. Public Health.

[17] Singh N., Hui D., Singh R., Ahuja I., Feo L., Fraternali F. (2017). Recycling of plastic solid waste: A state of the art review and future applications. Compos. Part B.

[18] Grigore M. (2017). Methods of recycling, properties and applications of recycled thermoplastic polymers. Recycling.

[19] Kazemi M., Kabir S., Fini E. (2021). State of art in recycling waste thermoplastics and thermosets and their applications in construction. Resour. Conserv. Recycl..

[20] Vogt B., Stokes K., Kumar S. (2021). Why is recycling of postconsumer plastics so challenging?. ACS Appl. Polym. Mater..

[21] Ragaert K., Delva L., Van Geem K. (2017). Mechanical and chemical recycling of solid plastic waste. Waste Manag..

[22] Maris J., Bourdon S., Brossard J.M., Laurent C., Fontaine L., Montembault V. (2018). Mechanical recycling: Compatibilization of mixed thermoplastic wastes. Polym. Degrad. Stab..

[23] Hopewell J., Dvorak R., Kosior E. (2009). Plastic recycling: Challenges and opportunities. Philos. Trans. R. Soc. B Biol. Sci..

[24] Cimpan C., Maul A., Jansen M., Pretz T., Wenzel H. (2015). Central sorting and recovery of MSW recyclable materials: A review of technological state-of-the-art, cases, practice and implications for materials recycling. Environ. Manag..

[25] Calero M., Martin-Lara M., Godoy V., Quesada L., Martinez D., Peula F., Soto J. (2018). Characterization of plastic materials present in municipal solid waste: Preliminary study for their mechanical recycling. Multidiscip. J. Waste Resour. Residues.

[26] Pandey J., Reddy R., Kumar P., Singh R.P. (2005). An overview on the degradability of polymer nanoncomposites. Polym. Degrad. Stab..

[27] Capone C., Di Landro L., Inzoli F., Penco M., Sartore L. (2007). Thermal and mechanical degradation during polymer extrusion processing. Polym. Eng. Sci..

[28] Lambert S., Sinclair C., Boxall A. (2014). Occurrence, degradation, and effect of polymer-based materials in the environment. Rev. Environ. Contam. Toxicol..

[29] He Y., Li H., Xiao X., Zhao X. (2021). Polymer degredation: Category, mechanism and development prospect. E3S Web Conferences ICGEC21.

[30] Harris M., Potgieter J., Archer R., Arif K. (2019). Effect of material and process specific factors on the strength of printed parts in Fused Filament Fabrication: A review on recent developments. Materials.

[31] Castro-Aguirre E., Iñiguez-Franco F., Samsudin H., Fang X., Auras R. (2016). Poly(lactic acid)–Mass production, processing, industrial applications, and End of Life. Adv. Drug Deliv. Rev..

[32] Murariu M., Dubois P. (2016). PLA composites: From production to properties. Adv. Drug Deliv. Rev..

[33] Nampoothiri K., Nair N., John R. (2010). An overview of the recent developments in polylactide (PLA) research. Bioresour. Technol..

[34] Jamshidian M., Tehrany E., Imran M., Jacquot M., Desobry S. (2010). Polylactid acid: Production, applications, nanocomposites, and release studies. Compr. Rev. Food Sci. Food Saf..

[35] Chacón J., Caminero M., García-Plaza E., Núnez P.J. (2017). Additive Manufacturing of PLA structures using Fused Deposition Modelling: Effect of process parameters on mechanical properties and their optimal selection. Mater. Des..

[36] Ilyas R., Sapuan S., Harussani M., Hakimi M., Haziq M., Atika M., Asyraf M., Ishak M., Razman M., Nurazzi N. (2021). Asrofi, Polylactic Acid (PLA) Biocomposite: Processing, Additive Manufacturing and advanced applications. Polymers.

[37] Sato H., Ogawa H. (2009). Review on development of Polypropylene manufacturing process. Materials.

[38] Maddah H. (2016). Polypropylene as a promising plastic: A review. Am. J. Polym. Sci..

[39] Dabbak S., Illias H., Ang B.C., Latiff N., Makmud M. (2017). Electrical properties of Polyethylene/Polypropylene compounds of high voltage insulation. Energies.

[40] Patil A., Patel A., Purohit R. (2017). An overview of polymeric materials for automotive applications. Mater. Today Proc..

[41] Antosik A., Kowalska U., Stobińska M., Dziecol P., Pieczykolan M., Kozlowska K., Bartkowiak A. (2021). Develepoment and characterization of bioactive Polypropylene films for food packaging applications. Polymers.

[42] Genis A. (2015). Analysis of the global and Russian markets for Polypropylene and of its main consumption areas. Russ. J. Gen. Chem..

[43] Jin M., Neuber C., Schmidt H.W. (2020). Tailoring polypropylene for extrusion-based Additive Manufacturing. Addit. Manuf..

[44] Carneiro O., Silva A., Gomes R. (2015). Fused Deposition Modelling with Polypropylene. Mater. Des..

[45] Vidakis N., Petousis M., Velidakis E., Tzounis L., Mountakis N., Kechagias J., Grammatikos S. (2021). Optimization of the filler concentration on Fused Filament Fabrication 3D printe polypropylene with titanium oxide nanocomposites. Materials.

[46] Kechagias J., Chaidas D., Vidakis N., Salonitis K., Vaxevanidis N. (2022). Key parameters controlling surface quality and dimensional accuracy: A critical review of FFF process. Mater. Manuf. Process..

[47] Kechagias J., Vidakis N., Petousis M., Mountakis N. (2022). A multi-parametric process evaluation of the mechanical response of PLA in FFF 3D printing. Mater. Manuf. Process..

[48] Das A., Riet J., Bortner M., McIlroy C. (2022). Rheology, crystallization, and process conditions: The effect on interlayer properties in three-dimensional printing. Phys. Fluids.

[49] Romani A., Rognoli V., Levi M. (2021). Design, materials, and extrusion-based Additive Manufacturing in Circular Economy contexts: From waste to new products. Sustainability.

[50] Despeisse M., Baumers M., Brown P., Charnley F., Ford S.J., Garmulewicz A., Knowles S., Minshall T.H.W., Mortara L., Reed-Tsochas F.P. (2016). Unlocking value for a Circular Economy through 3D printing: A research agenda. Technol. Forecast. Soc. Chang..

[51] Shanmugam V., Das O., Neisiany E., Babu K., Singh S., Hednqvist M., Berto F., Ramakrishna S. (2020). Polymer recycling in Additive Manufacturing an opportunity for the Circular Economy. Mater. Circ. Econ..

[52] Sanchez F.C., Boudaoud H., Carmago M., Pearce J. (2020). Plastic recycling and Additive Manufacturing: A systematic literature review and opportunities for the Circular Economy. J. Clean. Prod..

[53] Fico D., Rizzo D., Casciaro R., Corcione C.E. (2022). A review of polymer-based materials for Fused Filament Fabrication (FFF): Focus on sustainability and recycled materials. Polymers.

[54] Lanzotti A., Martorelli M., Maietta S., Gerbino S., Penta F., Gloria A. A comparison between mechanical properties of specimen 3D printed with virgin and recycled PLA. Proceedings of the 12th CIRP Conference on Intelligent Computation and Manufacturing Engineering (ICME18).

[55] Sanchez F.C., Boudaoud H., Hoppe S., Camargo M. (2017). Polymer recycling in an open-source Additive Manufacturing context: Mechanical issues. Addit. Manuf..

[56] Tanney D., Meisel N., Moore J. Investigating material degradation through the recycling of PLA in additively manufactured parts. Proceedings of the Annual International Solid Freeform Fabrication Symposium.

[57] Beltrán F., Arrieta M., Moreno E., Gaspar G., Muneta L., Carrasco-Gallego R., Yáñez S., Hidalgo-Carvajal D., de la Orden M., Urreaga J.M. (2021). Evaluation of the technical viability of distributed mechanical recycling of PLA 3D printing wastes. Polymers.

[58] Sasse J., Pelzer L., Schön M., Ghaddar T., Hopmann C. (2022). Investigation of recycled and coextruded PLA filament for Additive Manufacturing. Polymers.

[59] Spoerk M., Arbeiter F., Raguz I., Holzer C., Gonzalez-Gutierrez J. (2019). Mechanical recycability of Polypropylene composites produced by material extrusion-based Additive Manufacturing. Polymers.

[60] Vidakis N., Petousis M., Tzounis L., Maniadi A., Velidakis E., Mountakis N., Papageorgiou D., Liebscher M., Mechtcherine V. (2020). Sustainable Additive Manufacturing: Mechanical response of Polypropylene over multiple recycling processes. Sustainability.

[61] Dobránsky J., Pollák M., Behálek L., Svetlik J. (2021). Implementation of a recycled Polypropylene homopolymer material for use in Additive Manufacturing. Sustainability.

[62] Pickering K.L., Stoof D. (2017). Sustainable composite Fused Deposition Modelling filament using post-consumer recycled Polypropylene. J. Compos. Sci..

[63] Vidakis N., Petousis M., Maniadi A. (2021). Sustainable Additive Manufacturing: Mechanical response of high-density polyethylene over multiple recycling processes. Recycling.

[64] Momanyi J., Herzog M., Muchiri P. (2019). Analysis of thermomechanical properties of selected class of recycled thermoplastic materials based on their applications. Recycling.

[65] Jubinville D., Esmizadeh E., Saikrishnan S., Tzoganakis C., Mekonnen T. (2020). A comprehensive review of global production and recycling methods of polyolefin (PO) based products and their post-recycling applications. Sustain. Mater. Technol..

[66] Saikrishnan S., Jubinville D., Tzoganakis C., Mekonnen T. (2020). Thermomechanical degradation of polypropylene (PP) and low-density polyethylene (LDPE) blends exposed to simulated recycling. Polym. Degrad. Stab..

[67] Gudadhe A., Bachar N., Kumar A., Andrade P., Kumaraswamy G. (2019). Three-Dimensional Printing with waste High-Density Polyethylene. ACS Appl. Polym. Mater..

[68] Zander N., Gillan M., Burckhard Z., Gardea F. (2019). Recycled polypropylene blends as novel 3D printing materials. Addit. Manuf..

[69] Badia J.D., Amparo R. (2016). Mechanical recycling of Polylactide, upgrading trends and combination of valorisation techniques. Eur. Polym. J..

[70] Villaplana F., Karlsson S. (2009). Quality concepts for the improved use of recycled polymetric materials: A review. Macromol. Mater. Eng..

[71] Strömberg E., Karlsson S. (2009). The design of a test protocol to model the degradation of polyolefins during recycling and service life. J. Appl. Polym. Sci..

[72] (2016). Standard guide for accelerated aging of sterile barrier systems for medical devices.

[73] Garlotta D. (2001). A Literature Review of Poly(Lactic Acid). J. Polym. Environ..

[74] Lanyi F., Wenzke N., Kaschta J., Schubert D. (2020). On the determination of the enthalpy of fusion of α-crystalline isotactic polypropylene using differential scanning calorimetry, x-ray diffraction, and Fourier-Transform Infrared Spectroscopy: An old story revisited. Adv. Eng. Mater..

[75] Reyes-Labarta J., Olaya M., Marcilla A. (2006). DSC and TGA study of the transitions involved in the thermal treatment of binary mixtures PE and EVA copolymer with a crosslinking agent. Polymer.

[76] Cullis C., Hirschler M. (1981). The Combustion of Organic Polymers.

[77] León-Cabezas M., Martínez-Garcia A., Varela-Gandia F.J. (2017). Innovative functionalized monofilaments for 3D printing using Fused Deposition Modelling for the toy industry. Procedia Manuf..

[78] Cardona C., Curdes A., Isaacs A. (2016). Effects of filament diameter tolerances in Fused Filament Fabrication. IU J. Undergrad. Res..

[79] Spoerk M., Svandaiah C., Arbeiter F., Sapkota J., Holzer C. (2019). Optimization of mechanical properties of glass-spheres-filled Polypropylene composites for extrusion-based Additive Manufacturing. Polym. Compos..

[80] Menyhárd A. (2015). Direct correlation between modulus and the crystalline structure in isotactic polypropylene. Express Polym. Lett..

[81] El-Hadi A., Schnabel R., Straube E., Müller G., Henning S. (2002). Correlation between degree of crystallinity, morphology, glass temperature, mechanical properties and biodegradation of poly(2-hydroxalkonate) PHAs and their blends. Polym. Test..

[82] Meijer H., Govaert L. (2005). Mechanical performance of polymer systems. The relation between structure and properties. Prog. Polym. Sci..

[83] Shetty S., Shetty N. (2019). Investigation of mechanical properties and applications of polylactic acids—A review. Mater. Res. Express.

[84] Yu H., Huang N., Wang C., Tang Z. (2003). Modeling of poly(L-lactide) thermal degradation: Theoretical prediction of molecular weight and polydispersity index. J. Appl. Polym. Sci..

[85] Das A., Marnot A., Fallon J., Martin S., Joseph E., Bortner M. (2020). Material extrusion-based Additive Manufacturing with blends of polypropylene and hydrocarbon resins. ACS Appl. Polym. Mater..

[86] Bhushan B. (2000). Modern Tribology Handbook, Two Volume Set.

[87] Alsoufi M., Elsayed E. (2017). How surface roughness performance of printed parts manufactured by desktop FDM 3D printer with PLA is influenced by measuring direction. Am. J. Mech. Eng..

[88] Chaidas D., Kitsakis K., Kechagias J., Maropoulos S. (2016). The impact of temperature changing on surface roughness of FFF process. IOP Conf. Ser. Mater. Sci. Eng..

