# Supplementary material for: Thermo-Mechanical Recyclability of Additively Manufactured Polypropylene and Polylactic Acid Parts and Polypropylene Support Structures
Source: Polymers (Basel). 2023 May 12;15(10):2291. doi: 10.3390/polym15102291 (PMC10223719; doi:10.3390/polym15102291)
Supplement: Supplementary file 1 [file polymers-15-02291-s001.zip › polymers-2338097-supplementary.pdf]

Supporting Information

Thermo-mechanical recyclability of additively manufactured polypropylene and polylactic acid parts and polypropylene support structures

Niko Nagengast[1], Christian Bay[2,3], Frank Döpper[2,3], Hans-Werner Schmidt[4,5], Christian Neuber[4*]

1 Biomechanics, University of Bayreuth, Universitaetsstrasse 30, 95447 Bayreuth, Germany
2 Research Center for Additive Innovations, University of Bayreuth, Universitaetsstrasse 30, 95447 Bayreuth, Germany
3 Manufacturing and Remanufacturing Technology, University of Bayreuth, Universitaetsstrasse 30, 95447 Bayreuth, Germany
4 Macromolecular Chemistry, University of Bayreuth, Universitaetsstrasse 30, 95447 Bayreuth, Germany
5 Bavarian Polymer Institute, University of Bayreuth, Universitaetsstrasse 30, 95447 Bayreuth, Germany

* Correspondence: christian.neuber@uni-bayreuth.de

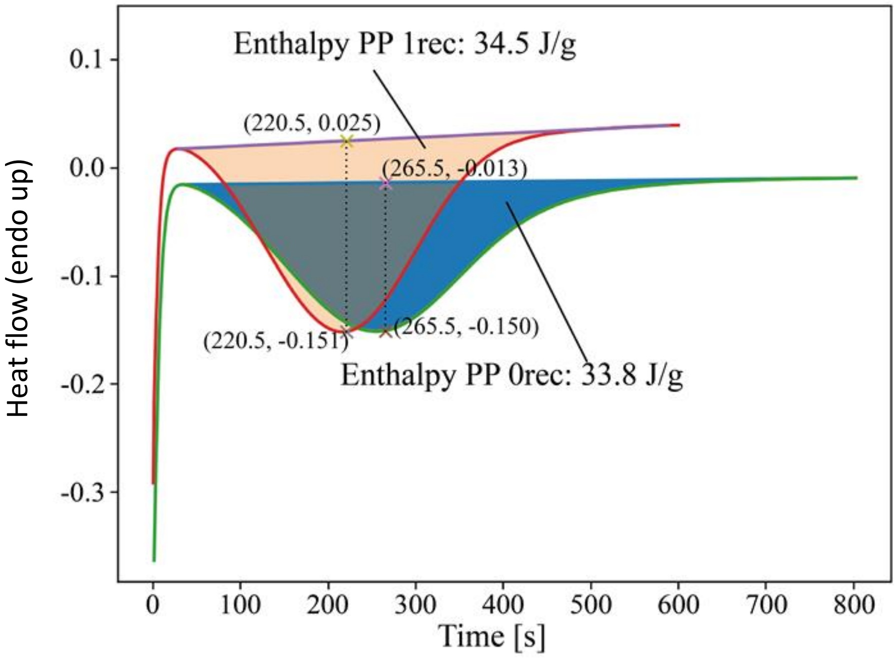

Figure S1: DSC curves of isothermal crystallization measurements of the virgin (PP 0rec) and recycled (PP 1rec) Polypropylene at T = 110°C.

Table S1: E-Modulus of the investigated polymers in 0°- direction

	**0° E-Modulus values** [MPa]					
	PLA 0rec	PLA 1rec	PP 0rec	PP 1rec	PP Sup 0rec	PP Sup 1rec
**MAX**	3538	3380	545	521	1479	1491
**MIN**	2483	2230	413	482	1430	1212
**MEAN**	2971	2836	445	500	1456	1401
**STDV**	312	377	47	20	21	70

Table S2: Tensile strain values of investigated polymers in 0°- direction

	**0° Tensile strain at break values** [%]					
	PLA 0rec	PLA 1rec	PP 0rec	PP 1rec	PP Sup 0rec	PP Sup 1rec
**MAX**	7.3	10.8	1856	1930	283	252
**MIN**	1.9	3.0	1553	1915	132	56
**MEAN**	3.6	6,0	1697	1929	199	163
**STDV**	1.7	2.4	88	8.5	58	66

Table S3: Tensile yield strenth values of investigated polymers in 0°- direction

	**0° Tensile yield strength values** [MPa]					
	PLA 0rec	PLA 1rec	PP 0rec	PP 1rec	PP Sup 0rec	PP Sup 1rec
**MAX**	56	57	18	19	18	19
**MIN**	45	47	14	16	15	13
**MEAN**	52	53	16	17	16	16
**STDV**	3.1	2.9	2.6	0.5	1.4	1.1

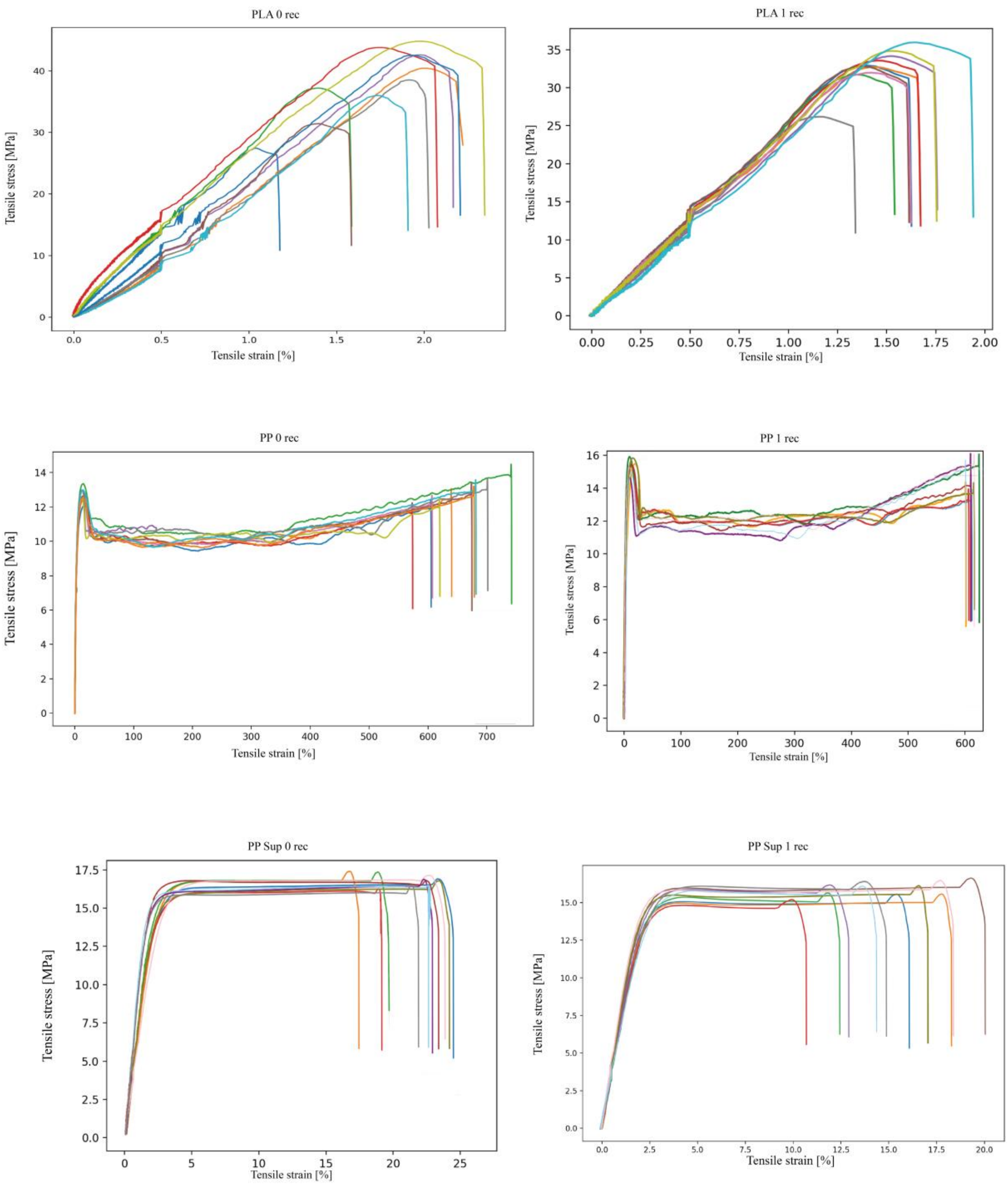

Figure S2: Tensile stress-strain curves of investigated polymers in 90°- direction.
